# Comparison of anaemia and parasitaemia as indicators of malaria control in household and EPI-health facility surveys in Malawi

**DOI:** 10.1186/1475-2875-9-107

**Published:** 2010-04-21

**Authors:** Don P Mathanga, Carl H Campbell, Jodi Vanden Eng, Adam Wolkon, Rachel N Bronzan, Grace J Malenga, Doreen Ali, Meghna Desai

**Affiliations:** 1 Malaria Alert Centre, College of Medicine, University of Malawi, P/Bag 360, Blantyre 3, Malawi; 2 Department of Community Health, College of Medicine, University of Malawi, Blantyre, Malawi; 3 Malaria Branch, Centers for Disease Control and Prevention, Atlanta, USA; 4 Ministry of Health, National Malaria Control Programme, Malawi

## Abstract

**Background:**

The World Health Organization has recommended that anaemia be used as an additional indicator to monitor malaria burden at the community level as malaria interventions are nationally scaled up. To date, there are no published evaluations of this recommendation.

**Methods:**

To evaluate this recommendation, a comparison of anaemia and parasitaemia among 6-30 month old children was made during two repeated cross-sectional household (HH) and health facility (HF) surveys in six districts across Malawi at baseline (2005) and in a follow-up survey (2008) after a scale up of malaria control interventions.

**Results:**

HH net ownership did not increase between the years (50.5% vs. 49.8%), but insecticide treated net (ITN) ownership increased modestly from 41.5% (95% CI: 37.2%-45.8%) in 2005 to 45.3% (95% CI: 42.6%-48.0%) in 2008. ITN use by children 6-30 months old, who were living in HH with at least one net, increased from 73.6% (95% CI:68.2%-79.1%) to 80.0% (95% CI:75.9%-84.1%) over the three-year period. This modest increase in ITN use was associated with a decrease in moderate to severe anaemia (Hb <8 g/dl) from 18.4% (95% CI:14.9%-21.8%) in 2005 to 15.4% (13.2%-17.7%) in 2008, while parasitaemia, measured as positive-slide microscopy, decreased from 18.9% (95% CI:14.7%-23.2%) to 16.9% (95% CI:13.8%-20.0%), a relative reduction of 16% and 11%, respectively. In HF surveys, anaemia prevalence decreased from 18.3% (95% CI: 14.9%-21.7%) to 15.4% (95% CI: 12.7%-18.2%), while parasitaemia decreased from 30.6% (95% CI: 25.7%-35.5%) to 13.2% (95% CI: 10.6%-15.8%), a relative reduction of 15% and 57%, respectively.

**Conclusion:**

Increasing access to effective malaria prevention was associated with a reduced burden of malaria in young Malawian children. Anaemia measured at the HF level at time of routine vaccination may be a good surrogate indicator for its measurement at the HH level in evaluating national malaria control programmes.

## Background

The unprecedented increase of investment in the control of malaria in Africa means that over the coming years, there should be a substantial reduction in malaria-related morbidity and mortality in these settings. Unfortunately, there are still few affordable and reliable indicators that can be used to measure short-term, malaria-specific impact of the scale-up of various interventions at the community level. Malaria related mortality, an important overall malaria control target, is difficult to diagnose, define and measure at the population level [1]. Its measurement is confined to health facilities - which capture only a small proportion of the malaria burden - and, at a population level, to selected, small-scale sentinel 'demographic surveillance' sites [2].

The World Health Organization (WHO) and Roll Back Malaria (RBM) Partnership have recommended that anaemia be used as an additional indicator to monitor malaria burden at the community level as interventions are nationally scaled-up [3]. This recommendation is based on results of an extensive review conducted by Korenromp *et al *(2004) showing that, in areas of stable malaria transmission, moderate-to-severe anaemia (haemoglobin <8 g/dl) is more sensitive than is parasite prevalence, and may respond more quickly than mortality to changes in malaria exposure due to increasing coverage of malaria interventions such as insecticide-treated bed nets (ITNs), malarial prophylaxis, and indoor residual spraying [4]. The review indicated that, in randomized controlled trials, the impact of ITNs on anaemia was more pronounced than on parasitaemia prevalence or clinical malaria.

Other reasons to include anaemia as an indicator for malaria control include: (a) its association with malaria in areas of high malaria transmission, (b) its ability to be measured and quantified in the field, (c) it has a morbidity burden, and (d) severe malarial anaemia, for young children and pregnant women, is on the pathway to mortality, which is the overall RBM target.

To date, there is no published literature evaluating this recommendation, or whether measurement of these indicators at time of routine immunizations could serve as a comparable substitute to measurement at the household level. This paper presents the utility of two approaches to monitoring and evaluation recommended in the WHO/RBM guidelines. The success of interventions implemented under the National Malaria Control Programme (NMCP) and the Roll Back Malaria Malawi country plan were assessed using both parasitaemia and anaemia. The evaluation was also designed to determine if measurement of these indicators at the health facility level at time of Expanded Programme on Immunization (EPI) visits could be a comparable substitute for measurement at the household level, information that is potentially very useful to Malawi and other malaria-endemic countries as it is logistically easier and cheaper to conduct health facility surveys than household surveys.

## Methods

### Study site

**Figure 1:**
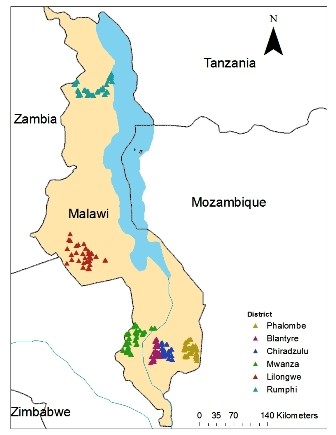
**Map of Malawi showing the 6 districts where the household survey in 2005 and 2008**.

The study consisted of two population based cross-sectional surveys conducted in six sentinel districts (Figure 1) across Malawi at baseline (April/May 2005) and follow-up (April/May 2008). The districts span the spectrum of malaria transmission intensity in Malawi, and each one is divided into enumeration areas (EAs), which generally range from 10-400 households. Both surveys were conducted at the end of the rainy season, when malaria-related anaemia reaches its peak. There were no significant differences in mean rainfall (previous 90 days) or maximum temperatures (previous 30 days) between the two survey periods. Each of the surveys had two components, a community-based household (HH) survey conducted in Blantyre, Mwanza, Phalombe, Chiradzulu, Lilongwe and Rumphi districts; and a health facility survey conducted at the EPI clinics (EPI-HF) in Blantyre, Lilongwe and Rumphi districts. In Malawi, the entire population is at risk of malaria and transmission is perennial peaking during the rainy season (November to April). Malaria is the leading cause of death and illness in under-five children and pregnant women [5]; infant and under-five mortality are estimated at 71/1,000 and 111/1,000 live births, respectively [6]. EPI coverage is >80% for targeted diseases [6].

**Figure 2:**
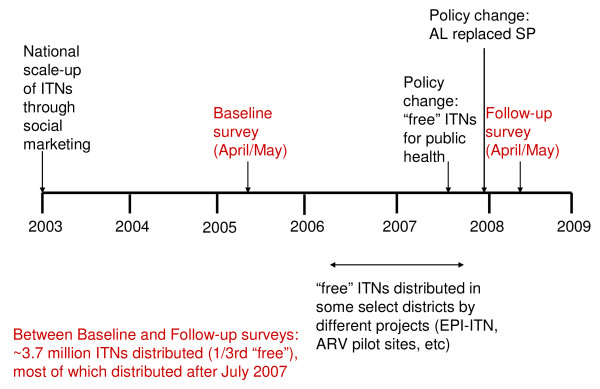
**Timeline of relevant malaria interventions in Malawi**.

Recognizing that malaria is a major public health problem, the government of Malawi is focusing on the rapid scale up of malaria interventions so as to significantly reduce malaria morbidity and mortality in the country (Figure 2). Three strategic areas identified for scale-up include case management, intermittent preventive treatment in pregnant women (IPTp) and use of ITNs [7]. Malawi changed its treatment policy to artemisinin combination therapy (ACT) in December 2007, with the first-line treatment for uncomplicated malaria being artemether-lumefantrine and the second-line artesunate-amodiaquine. Currently, malaria is clinically diagnosed and free malaria treatment can only be sought from public health facilities. In 2006, only 21% of the children with fever were promptly treated with an effective anti-malarial, against a national target of 80% [6]. For vector control, heavily subsidized ITNs are targeted at under-five children and pregnant women through health facilities and limited time mass campaigns. Free ITNs for public health distribution were not implemented until July 2007. None of the districts involved in this study had an indoor residual spraying (IRS) programme. As a result of planned intensified national scale-up of malaria interventions, between 2005 and 2008, it was expected that coverage with malaria interventions would significantly increase between the two surveys.

The study protocol was approved by Institutional Review Boards at the University of Malawi and the US Centers for Disease Control and Prevention.

### Sampling

For the 2005 HH survey, 30 enumeration areas (EAs) were selected with probability proportional to estimated size (PPS) in each of the six districts. The sampling frame was developed through the use of global positioning system (GPS) linked with hand-held computers (PDAs) to allow rapid preliminary mapping of the EA from which a simple random sample of households was selected. This method involved: a) mapping every household in the selected EA using PDAs equipped with GPS, b) selecting a random sample of 11 households, and c) using the GPS to navigate back to the households and perform the interview [8]. This translates to 330 households per district, or 1,980 households for all six districts. All selected households were invited to participate in the survey during which a questionnaire was administered collecting data on household economic status and net ownership/use, and children 6-30 months of age providing a blood sample for determination of parasitaemia and haemoglobin.

In the 2008 HH survey, a modified EPI cluster survey method, as described by Turner, was used to select households [9]. A different method was used because of logistical difficulties encountered in the mapping and listing of households in many of the EAs in the 2005 HH survey. The modified cluster survey method involved: a) selection of the same EAs as the ones used in the 2005 HH survey, b) using EA maps which list the expected number of households and the distribution of the households on the maps (with field observation including confirmation of EA borders), (c) creating sub-clusters or segments of approximately equal size, d) randomly selecting one segment, and e) interviewing all households in the selected segment and obtaining blood samples from children 6-30 months of age. Listings were not needed because all HHs in the segment were included and the probability of selection was known.

The EPI-HF survey component was conducted in three of the six districts selected for the HH survey (Lilongwe, Blantyre, and Rumphi) immediately following the respective HH surveys in those districts. In each of the three districts, government health facilities, which provide EPI services, were chosen to attain a sample size of 540 children per district. Due to differences in enrolment rates, five health facilities were chosen in Blantyre, three in Rumphi and four in Lilongwe in order to meet the required sample size. The same health facilities were used in both the 2005 and 2008 surveys. Children 6-30 months of age attending the EPI clinic for a routine visit were systematically sampled from the clinic and invited to enrol.

### Sample size

Sample size was calculated based on assumption that 21% of all children 6-30 months old in the baseline survey would have Hb <8 g/dl (based on results from a 2003 household survey in Blantyre district). Sample sizes were calculated using 95% confidence limits, 80% power, and 10% adjustment for non-response to detect a 33% reduction in the prevalence of Hb <8 g/dl between two cross-sectional surveys. Using this estimate, a sample size of 1,900 households (712 children) was needed for the HH survey assuming 37% of households had children 6-30 months of age (DHS 2000) and a design effect of 1.32. A sample size of 1,617 children was required for the EPI-HF survey assuming a design effect of 3.0. The same sample size was applied for the 2005 and 2008 surveys.

### Data collection

#### Interview with parent/guardian

Structured interviews were conducted with household heads and/or parent/guardian of all children in selected households. Information was gathered on ownership of household assets, household net ownership, net use and health of children under five years of age; blood smears and haemoglobin results were obtained for children 6-30 months of age. Per national policy, children with an Hb<8 g/dl, or fever, or a history of fever in the previous two weeks, were treated with sulphadoxine-pyrimethamine (SP) (2005) or with artemether-lumefantrine (CoArtem^®^) (2008). All children with anaemia were treated with iron and albendazole as per national guidelines.

The questionnaire was designed in English and translated into two main languages spoken in the selected districts, namely Chichewa and Chitumbuka. Data were collected electronically using a PDA-based questionnaire developed using Visual CE 9.0 (Syware Inc., Cambridge, MA). During the survey, data were backed up onto a secure digital card every evening. Upon survey completion, PDAs were returned to Blantyre, where data were aggregated into a Microsoft Access database.

#### Laboratory procedures

Blood samples were collected from each child for on-the-spot measurement of haemoglobin concentration *(HemoCue, Angelhom, Sweden). *Thick blood smears were prepared for microscopic diagnosis of malaria parasites. Blood slides were stained with field stain and read by an expert microscopist at the College of Medicine. Slides were declared negative if no asexual parasites were found after examining 100 high-power fields. Slide reading was blinded and the external quality control consisted of having all positive slides and 10% of randomly selected negative slides read again by an independent microscopist.

#### Definitions

*Household *was defined as a family, comprising the head of household (man or woman), husband or wife, children and immediate family members who shared income. An ITN was defined as any bed net that had been treated with insecticide in the previous 12 months or a long-lasting insecticidal net (a net treated during the manufacturing process). Adherence to net/ITN was defined as a child sleeping under a net/ITN the previous night in a HH that owns a net/ITN. Parasitaemia was defined as the presence of any asexual malaria parasites detected on a thick peripheral blood smear. A child with haemoglobin concentration 5 to <8 g/dL was classified as moderately anaemic, while those with haemoglobin <5 g/dL were classified as severely anaemic. For the purpose of analysis, anaemia was defined as any haemoglobin level <8 g/dL. Clinical malaria was defined as a documented axillary temperature ≥ 37.5°C in the presence of any asexual parasitaemia. Household wealth scores were developed using loading factors from the 2004 Malawi Demographic and Health Survey [5]. This is a composite score obtained from a principle components model, including variables assessing household assets (electricity, bike, radio, type of floor material and toilet, source of drinking water, etc). These wealth scores were partitioned to create wealth quintiles.

### Statistical analysis

Estimates of frequencies were calculated using SAS survey procedures (SAS v9.1.3) which use a Taylor series expansion to account for stratification, cluster sampling and unequal probabilities of selection. All analyses of HH data used weights equal to the inverse probability of selection. The EPI-HF survey was not a probability sample and could not be weighted. The outcome variable anaemia was considered as dichotomous (Hb < 8 g/dL or Hb >= 8 g/dL) and parasitaemia was measured as presence or absence of asexual parasites.

Multivariate logistic regression models were performed in SAS using Generalized Estimating Equations (GEE) with an exchangeable working correlation matrix to produce empirically corrected standard error estimates that account for clustering and unequal selection probabilities. Rao-Scott chi-square values were used to detect associations between the outcome variable and the predictors of anaemia such as net use, ITN use, and prompt and appropriate treatment of fever. To observe whether there were any statistically significant differences between HH and EPI-HF measurement in change of anaemia from 2005 and 2008, an interaction term between study year and an indicator variable identifying the survey type (HH or EPI-HF) was created. The final models for anaemia and parasitaemia were adjusted for age, ethnicity, SES, clinical malaria (in the case of anaemia), current fever (for parasitaemia), district, and whether or not the child had slept under an ITN the previous night.

## Results

The 2005 HH survey approached 1,739 HHs for participation over a four-week period, and recruited 926 children between the ages of 6-30 months; immediately following the HH survey, 12 EPI-HFs were surveyed over a two-week period to yield 1,637 children in this same age group. Children included in the HH survey were older, had a different distribution of ethnic groups, were less likely to have sought treatment within 24 hrs, and were less likely to have concurrent fever than children included in the EPI-HF survey (see Additional file 1, Table A: Characteristics of children participating in the 2005 and 2008 household and EPI-health facility surveys).

In 2008, 10,034 HHs were approached for participation, and 4,565 children between the ages of 6-30 months were recruited; the 12 EPI-HFs selected in 2005 were re-surveyed to yield 1,909 children in this same age group. Children included in the 2008 HH survey were poorer, older, of different ethnic distribution, less likely to have slept under a net or an ITN the previous night, more likely to have reported a history of fever in the past two weeks, and less likely to have sought treatment within 24 hrs than children included in the EPI-HF survey in 2008 (See Table A, additional file 1).

**Figure 3:**
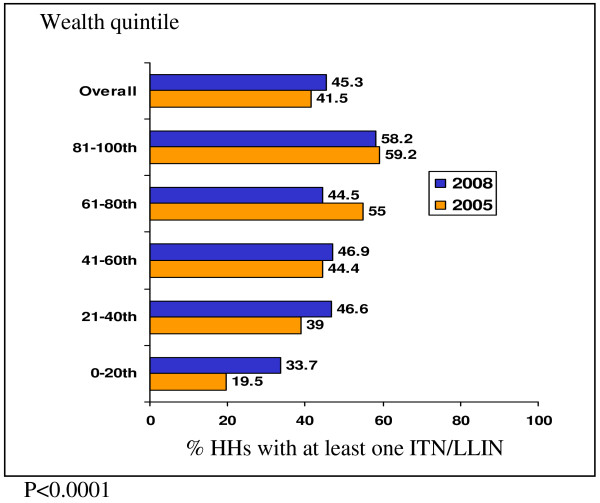
**ITN ownership by wealth index in HH surveys**.

Between the 2005 and 2008 HH surveys, there were some significant differences in characteristics of recruited children. The 2005 survey represented a higher proportion of HHs in the 3^rd ^wealth quintile and lower proportion in the 5^th ^(wealthiest) quintile than the 2008 survey. Overall, HH net ownership (regardless of treatment status) did not increase between the years (50.5% vs. 49.8%), but ITN ownership increased modestly from 41.5% (95% CI: 37.2%-45.8%) in 2005 to 45.3% (95% CI: 42.6%-48.0%) in 2008; ITN ownership was significantly increased in the poorest quintile (Figure 3). Among children <5 years old, 36.9% (95% CI: 31.5%-42.3%) and 41.5% (95% CI: 38.2%-44.7%) slept under an ITN the night before in the 2005 and 2008 surveys respectively, and approximately three out of four children adhered to ITNs in both years. There was a statistically significant increase in adherence to use of any net among children <5 years old (from 69.2% (95% CI: 63.5%-75.0%) to 80.5% (95% CI: 77%- 83.9%)). The 2005 survey also had significantly fewer children belonging to the Chewa ethnic group and higher proportion in the Tumbuka group than in the 2008 survey. Both history of fever and treatment seeking for fever were significantly reduced between the two survey years.

Between the 2005 and 2008 EPI-HF surveys, there was a significant increase from 36.7% [95% CI: 31.1% - 42.4%] to 49.4% [95% CI: 44.4% - 54.4%] in the proportion of children who reported sleeping under an ITN the previous night. Both the reported history of fever and current fever at the time of the survey were halved.

### Measuring change in anaemia and parasitaemia

In HH surveys, the prevalence of anaemia decreased from 18.4% (95% CI: 14.9%-21.8%) in 2005 to 15.4% (95% CI: 13.2%-17.7%) in 2008, while parasitaemia prevalence, measured by microscopy, decreased from 18.9% (95% CI: 14.7%-23.2%) to 16.9% (95% CI: 13.8%-20.0%), a relative reduction in crude prevalence from baseline of 16% and 11%, respectively. In EPI-HF surveys, anaemia decreased from 18.3% (95% CI: 14.9%-21.7%) to 15.5% (95% CI: 12.7%-18.2%), while malaria decreased from 30.6% (95% CI: 25.7%-35.5%) to 13.2% (95% CI: 10.6%-15.8%), a relative reduction of 15% and 57%, respectively (Table 1).

**Table 1:** Reduction in the crude prevalence of anaemia and parasitaemia among children 6-30 months of age in household and EPI-health facility surveys conducted in 2005 and 2008

	Household (HH) survey			Health Facility (HF) surveys		
	Baseline (2005)	Follow-up (2008)	Relative reduction ^#^**(%)**	Baseline (2005)	Follow-up (2008)	Relative reduction
Prevalence of anaemia (Hb<8 g/dl) in children 6-30 months;						
n/N	184/926	649/4461		299/1636	295/1909	
% (95% CI)*	18.4% (14.9-21.8)	15.4% (13.2-17.7)	16.1%(11 .4-18.8)	18.3% (14.9-21.7)	15.5% (12.7-18.2)	15% (14.8-16.1)
Prevalence of parasitaemia in children 6-30 months;						
n/N	195/799	607/4377		464/1516	247/1871	
% (95% CI)*	18.9% (14.7-23.2)	16.9% (13.8-20.0)	10.7% (6.1-13.8)	30.6% (25.7-35.5)	13.2% (10.6-15.8)	57% (55.5-58.8)

### Factors associated with anaemia and parasitaemia

Table 2 and Table 3 show association of anaemia and parasitaemia with age, household and clinical characteristics for each of the surveys. Gender, receipt of prompt treatment for fever, or number of children under the age of five living in the household were not significantly associated with anaemia or parasitaemia in any of the surveys.

**Table 2:** Association of anaemia and parasitaemia with household and clinical characteristics among children 6-30 months of age enrolled into the 2005 HH and EPI-HF surveys (adjusted for clustering)

2005	Household surveys (N = 926)				EPI-Health facility surveys (N = 1637)			
	Anaemia (Hb<8 g/dl) n (%)	χ2 P-value	Parasitaemia n (%)	χ2 P-value	Anaemia (Hb<8 g/dl) n (%)	χ2 P-value	Parasitaemia n (%)	χ2 P-value
Age (months)								
6-8	42 (28.0)	0.05	31 (25.6)	0.45	77 (21.0)	0.07	108 (31.3)	0.85
9-11	26 (13.9)		22 (14.6)		55(17.8)		88 (30.3)	
12-17	50 (19.4)		48 (21.5)		105(20.8)		138 (29.6)	
18-23	37 (16.3)		48 (16.3)		34(12.4)		74 (29.0)	
24-29	29 (15.1)		46 (17.1)		28(15.6)		56 (35.0)	
District								
Phalombe	47 (22.1)	<.0001	51(27.8)	<.0001		0.06	-	<.0001
Blantyre	13 (19.8)		15(14.9)		118(22.3)		52 (11.1)	
Chiradzulu	18 (20.2)		15(20.5)		-		-	
Mwanza	36 (28.3)		61(50.4)		-		-	
Lilongwe	37 (33.0)		30(42.0)		108 (19.7)		307 (58.9)	
Rumphi	33 (11.1)		23(7.82)		73 (13.1)		105 (19.9)	
Wealth quintile								
Poorest	48 (23.2)	0.60	53 (23.5)	0.16	80(28.9)	<.000	79 (30.6)	0.02
2^nd^	44 (18.4)		47 (22.4)		75(22.5)	1	133 (42.1)	
3^rd^	48 (17.4)		45 (14.2)		49(16.5)		91 (32.7)	
4^th^	33 (17.5)		40 (20.6)		49(14.5)		80 (26.0)	
Wealthiest	11 (13.1)		10 (9.04)		46(11.8)		81 (22.8)	
History of fever								
Yes	104 (24.0)	0.002	109 25.3)	0.003	144(22.8)	0.004	173 (30.2)	0.87
No	80 (14.3)		86 (14.1)		155(15.4)		291 (30.8)	
Currently febrile								
Yes	17 (26.0)	0.08	16 (34.3)	0.03	56 (29.3)	0.001	68 (40.0)	0.02
No	153 (16.2)		179(18.1)		243 (16.8)		395 (29.4)	
Clinical malaria								
Yes	7 (48.1)	.006	NA		21 (30.9)	.006	NA	
No	138 (15.4)				260 (18.0)			
Slept under a net the previous night								
Yes	56 (12.9)	0.002	63 (14.1)	0.04	102 (15.2)	0.03	167 (27.1)	0.06
No	128 (22.9)		132(23.1)		197 (20.4)		297 (33.0)	
Slept under ITN previous night								
Yes	53 (14.0)	0.03	54 (13.9)	0.05	89 (14.8)	0.01	155 (27.9)	0.18
No	131 (21.5)		141 22.5)		210 (20.3)		309 (32.2)	

**Table 3:** Association of anaemia and parasitaemia with household and clinical characteristics among children 6-30 months of age enrolled into the 2008 HH and EPI-HF surveys (adjusted for clustering).

2008	Household surveys (N = 4565)				EPI-Health facility surveys (N = 1909)			
	Anaemia (Hb<8 g/dl) n (%)	χ2 P-value	Parasitaemia n (%)	χ2 P-value	Anaemia (Hb<8 g/dl) n (%)	χ2 P-value	Parasitaemia n (%)	χ2 P-value
Age(months)								
6-8	95 (14.8)	0.19	65(13.8)	0.29	71 (15.3)	0.67	47 (10.4)	0.08
9-11	97 (20.8)		70(19.1)		66 (16.8)		50 (13.0)	
12-17	192 (15.0)		169(14.8)		87 (16.4)		67 (12.8)	
18-23	143 (15.4)		154(18.4)		47 (13.9)		49 (14.9)	
24-29	122 (13.6)		149(18.6)		24 (13.0)		34 (18.7)	
District								
Phalombe	179 (17.7)	0.01	193(81.4)	<.0001	-	0.07	-	<.0001
Blantyre	65 (11.3)		36(93.5)		73 (12.1)		59 (9.8)	
Chiradzulu	112 (18.8)		76(87.0)		-		-	
Mwanza	80 (13.2)		105(82.0)		-		-	
Lilongwe	127 (17.5)		165(76.6)		125 (19.7)		149 (24.7)	
Rumphi	86 (8.5)		32(96.0)		97 (14.4)		39 (5.8)	
Wealth quintile								
Poorest	168 (19.1)	<.0001	168(22.5)	<.0001	43 (21.6)	<.000	40 (20.4)	<.0001
2^nd^	155 (19.1)		141(18.5)		89 (19.1)	1	92 (20.3)	
3^rd^	161 (15.2)		170(20.4)		75 (19.5)		53 (14.1)	
4^th^	113 (13.9)		88(11.3)		50 (13.4)		36 (9.9)	
Wealthiest	52 (6.7)		40(7.6)		38 (7.9)		26 (5.4)	
History of fever								
Yes	229 (25.9)	<.0001	173(23.1)	<.0001	78(22.7)	0.001	63 (18.8)	<.0001
No	420 (11.5)		434(14.6)		217(13.9)		184 (12.0)	
Currently febrile								
Yes	95 (43.1)	<.0001	94 (51.0)	<.0001	22 (25.0)	0.04	37 (44.0)	<.0001
No	554 (13.7)		513 (14.8)		273(15.0)		210 (11.8)	
Clinical malaria								
Yes	59 (58.6)		NA		17 (45.9)	<.000	NA	
No	581 (14.2)	<.0001			270(14.7)	1		
Slept under a net the previous night								
Yes	235 (13.4)	0.03	211(14.1)	0.02	154(14.4)	0.20	99 (9.4)	0.0002
No	414 (16.8)		396(18.8)		141(16.7)		148(18.0)	
Slept under ITN previous night								
Yes	186(13.3)	0.03	179(14.6)	0.06	142(15.1)	0.68	90 (9.7)	0.002
No	463(16.7)		428(18.2)		153(15.8)		157(16.6)	

**Figure 4:**
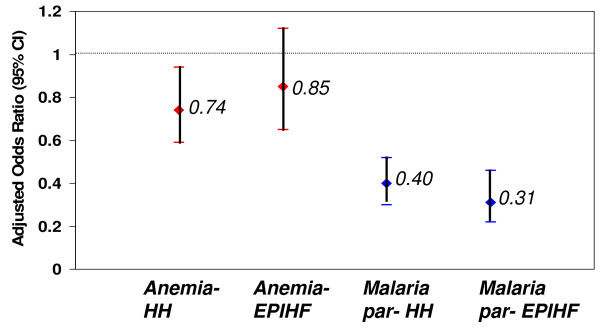
Effect of survey type on change in anaemia and parasitaemia between 2005 and 2008*. *adjusted for age, ethnicity, wealth, clinical malaria (in the case of anaemia), current fever (for parasitaemia), district, and whether or not the child had slept under an ITN the previous night.

Table 4 shows results of both unadjusted and adjusted logistic regression analyses of the association of anaemia and parasitaemia with type of survey (HH vs. EPI-HF) and year (2005 vs. 2008). Overall, there was no significant effect of type of survey on measured change in anaemia or parasitaemia over the 3 years. Adjusted analyses revealed that the odds of having anaemia in the 2008 HH survey was 0.74 (95% CI: 0.59-0.94) times that of the 2005 HH survey. The odds of having anaemia in the 2008 EPI-HF survey was 0.85 (95% CI: 0.65-1.12) times that of the 2005 EPI-HF survey. The reduction in the odds of having parasitaemia were even more pronounced and not significantly different between the two types of surveys (HH survey: 0.40 (95% CI: 0.3-0.52), EPI-HF survey: 0.31 (95% CI: 0.22-0.46)) (Figure 4).

**Table 4:** Association of survey type (HH vs. EPI-HF) and year (2005 vs. 2008) with prevalence of anaemia and parasitaemia among children 6-30 months of age in six districts in Malawi

	Anaemia		Parasitaemia	
	Unadjusted	Adjusted^a^	Unadjusted	Adjusted^b^
	OR (95% CI)	OR (95% CI)	OR (95% CI)	OR (95% CI)
	P* = 0.36	P* = 0.45	P* = 0.16	P* = 0.17
2005 HH	Reference	Reference	Reference	Reference
2005 EPI-HF	0.90 (0.67-1.22)	1.04 (0.77-1.40)	1.37 (0.90-2.07)	1.3 (0.9-1.95)
2008 HH	0.69 (0.55-0.85)	0.74 (0.59-0.94)	0.50 (0.41-0.61)	0.4 (0.3-0.52)
2008 EPI-HF	0.74 (0.55-0.99)	0.88 (0.65-1.20)	0.47 (0.33-0.67)	0.4 (0.3-0.56)

^a ^adjusted for age, ethnicity, wealth, clinical malaria, slept under ITN the previous night, and district

^b ^adjusted for age, ethnicity, wealth, current fever, slept under ITN the previous night, and district

* type 3 p-value for interaction between type of survey (HH vs. EPI-HF) and year (2005 vs. 2008)

## Discussion

The government of Malawi is rapidly scaling up a full range of existing evidence-based malaria prevention and control strategies. These strategies include use of ITNs, IPTp and prompt and effective case management, particularly among children who are sick with malaria. Between 2005 and 2008, 3,727,330 nets (PSI, personal communication) were distributed and a new malaria treatment policy was implemented countrywide, with artemether-lumefantrine replacing SP as the first-line drug. A modest, non-statistically significant increase in ITN household ownership (from 41.5% to 45.3%) and a statistically significant increase in adherence to any net (regardless of treatment status) were observed between 2005 and 2008. The results also show a moderate reduction in the odds of anaemia; the prevalence of anaemia decreased by a modest 15-16% from baseline, depending on the type of survey. In light of these data, and the very recent introduction of ACT, it can be argued that anaemia is a sensitive indicator to the scale up of malaria interventions and may thus be used as a proxy indicator to track the burden of malaria and demonstrate timely impact of malaria interventions in areas of high malaria This finding is important because existing impact indicators are either hard to measure at the national scale (e.g. all-cause under-five mortality, malaria-attributed mortality) or not sensitive enough to changes in malaria transmission (e.g. parasite prevalence). In contrast, anaemia is measurable and quantifiable in the field with the portable HemoCue haemoglobinometre using small volumes of capillary blood, it requires a smaller sample size, and gives more precise results than does measurement of all-cause mortality for children under-five years of age. However, anaemia may have limited utility in areas of lower malaria transmission where the attributable fraction of anaemia due to malaria is too low to reflect changes in malaria transmission/control.

Parasitaemia, another common indicator used to assess malaria control, was also evaluated in this study. The prevalence of parasitaemia was reduced by 11% in the HH survey and 57% in the EPI-HF survey. This observation supports the finding above that reduction in both anaemia and parasitaemia could have been due to the modest scale up of malaria interventions. The dramatic reduction in parasitaemia observed in the EPI-HF survey was unexpected but could be due to accidental inclusion of some sick children during the EPI-HF survey in 2005. This is supported by the data showing that the proportion of children who had fever at the time of recruitment into the EPI-HF survey was significantly decreased from 11.7% to 4.6% between 2005 and 2008 (p < 0.0001). A corresponding decrease was also observed in children presenting to the clinic with a history of fever in the past 48 hours (p < 0.0001)(see Table A, additional file 1). Adjusted analyses revealed that the reductions in the odds of parasitaemia in both types of surveys were not significantly different from each other.

Not surprisingly, the reduction in the prevalence of anaemia and parasitaemia observed in this study was very modest. A similar household evaluation conducted among children under five years of age in Zambia has reported over 50% reduction in both parameters between 2006 and 2008, likely due to an increase of >20% in household ownership of at least one mosquito net following a mass distribution campaign [10]. Household ITN ownership in the Malawian study only increased from 41.5% to 45.3% (p = 0.10) during the three-year interval. This modest and non-significant increase could be explained by several factors. First, between 2007 and 2008, the procurement and distribution of ITNs experienced a lot of logistical problems resulting in fewer subsidized nets being delivered to the target groups (PSI, personal communication). As a result, most of the ITNs during this period were only distributed after the 2008 follow up surveys. Secondly, there is the possibility that because the distribution of ITNs was targeted to only households with pregnant women or children aged less than five years, during the period between the two surveys, the same households were reached with ITNs as a result of the targeted distribution. This is supported by the fact that overall net ownership did not increase during this period. Population growth could also explain the modest increase in household ITN increase. While the number of new nets distributed is known, population growth in the communities surveyed is not known with a possibility that fewer nets were distributed compared to population growth. This failure to significantly scale up household ownership of ITNs has been reported in other surveys [6]. In this survey, in households with at least one ITN, over 75% of the children slept under an ITN suggesting good adherence to nets among children under five as has been reported elsewhere [11]. Therefore, in Malawi, more needs to be done to achieve the Roll Back Malaria target of protecting 80% of the population at risk by 2010. Rapid scale up of the ITN programme through mass distribution to achieve universal coverage could significantly reduce malaria morbidity, and especially malaria-related anaemia.

This study also shows that the reduction in anaemia, as measured during EPI-HF surveys, was similar to the reduction measured during the household surveys. Thus, anaemia measured at the EPI clinics at the facility level may be a good surrogate indicator of anaemia measurement at the household level. This finding has important programmatic implication because EPI-HF surveys have the advantage of being cheaper and logistically simpler to execute than household surveys. In addition, measurement of anaemia in the EPI clinic would be only a small burden for health workers since surveys would only be done periodically. The ability to measure the impact of malaria interventions through EPI clinics could afford District Health Management Teams the means to quickly evaluate the impact of the malaria programme and inform the efficient deployment of malaria interventions within the district.

The results of this evaluation should be interpreted in the context of the limitations of the study. The causes of anaemia are multifactorial; paediatric HIV infection and malnutrition are prominent ones [12-14]. As a result the reduction in the prevalence of anaemia observed in this study could be affected by improvements in coverage of non-malaria related interventions. This study did not collect information on other competing causes of anaemia, such as nutritional status, HIV, hookworm infection, helminths, or bacteraemia. However, between 2005 and 2008, there were no large scale nutritional interventions or other child health interventions that underwent rapid scale up in the selected districts which could have led to the decrease in the prevalence of anaemia. Children in the household surveys were significantly older than children in the EPI-health facility surveys (p < 0.0001). This difference in age may have introduced bias in the results; however, this is unlikely as the prevalence of anaemia in the EPI-HF survey compared very well to that in the household survey. Despite these limitations, it is reasonable to argue that the very modest decline in anaemia prevalence was as a result of the small increase in malaria control interventions, specifically the scale up of ITN coverage and the introduction of ACT, since other proxies for malaria such as fever in the last two weeks were also significantly reduced between 2005 and 2008.

The sampling strategies for the two household surveys were different from one another. In 2005, a simple random sample of households in each EA was selected, whereas in 2008, information was collected from all households in a randomly selected segment of each EA. Although both strategies have been validated [8,9], both strategies experienced challenges in the application of the respective techniques because of the physical size of EAs in Malawi. If some households were inadvertently missed due to these challenges, it may result in a bias in the estimates presented here. The generalizability of these results is also limited by the fact that the selection of districts, although geographically representative of the different transmission strata in Malawi, was a convenience sample. Furthermore, the EPI-HF survey was restricted to only three districts due to financial and logistical constraints.

## Conclusion

The results from this study suggest that anaemia may continue to be included as an important indicator for monitoring and evaluation in areas of moderate to high malaria transmission where the attributable fraction of anaemia due to malaria is high enough to reflect changes in malaria transmission and control. Anaemia (and maybe parasitaemia) measured at time of routine immunizations may be a good surrogate indicator for its measurement at the household level, and should be evaluated/verified in other settings.

## Competing interests

The authors declare that they have no competing interests.

## Authors' contributions

DPM was responsible for the design of the study, implementation of the study protocol, data analysis/interpretation, drafting of the manuscript and critical revision of the manuscript. CHC was responsible for the study concept, design and interpretation of the data. JVE was responsible for the statistical analysis/interpretation of the data and drafting of the manuscript. AW, RB, GJM and DA were responsible for implementing the protocol, supervision of the data collection and critical revision of the manuscript. MD was responsible for the study concept/design, analysis/interpretation of the data and revision of the manuscript. All authors read and approved the final manuscript.

## Supplementary Material

Additional file 1 **Table A. Characteristics of children participating in the 2005 and 2008 household and EPI-health facility surveys**. The table demographic information on children recruited during the 2005 and 2008 surveys. Click here for file
